# A Cross-Lingual Mobile Medical Communication System Prototype for Foreigners and Subjects with Speech, Hearing, and Mental Disabilities Based on Pictograms

**DOI:** 10.1155/2017/4306416

**Published:** 2017-11-02

**Authors:** Krzysztof Wołk, Agnieszka Wołk, Wojciech Glinkowski

**Affiliations:** ^1^Department of Multimedia, Polish-Japanese Academy of Information Technology, Koszykowa 86, 02-008 Warsaw, Poland; ^2^Department of Medical Informatics and Telemedicine, Medical University of Warsaw, Warsaw, Poland

## Abstract

People with speech, hearing, or mental impairment require special communication assistance, especially for medical purposes. Automatic solutions for speech recognition and voice synthesis from text are poor fits for communication in the medical domain because they are dependent on error-prone statistical models. Systems dependent on manual text input are insufficient. Recently introduced systems for automatic sign language recognition are dependent on statistical models as well as on image and gesture quality. Such systems remain in early development and are based mostly on minimal hand gestures unsuitable for medical purposes. Furthermore, solutions that rely on the Internet cannot be used after disasters that require humanitarian aid. We propose a high-speed, intuitive, Internet-free, voice-free, and text-free tool suited for emergency medical communication. Our solution is a pictogram-based application that provides easy communication for individuals who have speech or hearing impairment or mental health issues that impair communication, as well as foreigners who do not speak the local language. It provides support and clarification in communication by using intuitive icons and interactive symbols that are easy to use on a mobile device. Such pictogram-based communication can be quite effective and ultimately make people's lives happier, easier, and safer.

## 1. Introduction

Some authors have identified the potential of mobile devices for application to medical needs and the health domain. Wac [1] surveyed the literature on a variety of smartphone-related technologies that make personalized health information and services widely available. She identified smartphones as a key, emergent trend. However, she indicated that significant progress is needed in applying smartphones to clinical medical practice. In addition, the needs of people with hearing impairments, in particular, are recognized as another area in which research is needed [2].

Mobile devices are highly accessible and usable today. Susick [3] reviewed the literature on the potential for smartphones to improve mental health care. Most study participants were able to use a smartphone, and participating care providers were able to collect the data needed to deliver care via phone. This survey suggests the potential for mobile devices to lower barriers and improve the delivery of mental health care.

Luxton et al. [4] also observed the wide availability of mobile devices. In particular, they noted that two-way communication via smartphones has vast potential for medical applications, especially for telemental health care. In addition to accessibility, this could improve healthcare quality and outcomes. However, they noted that few applications have applied mobile technologies to such care.

**Figure 1:**
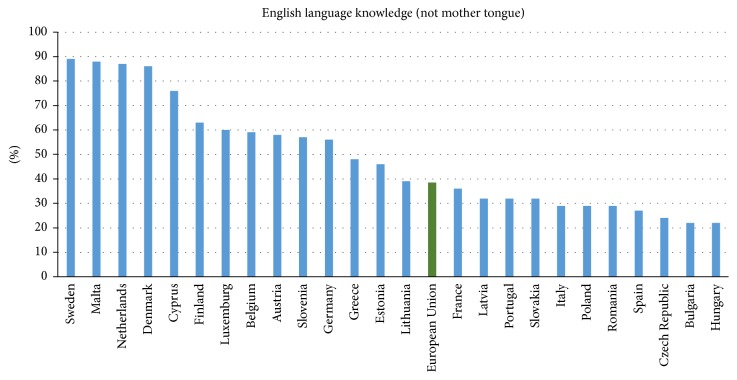
Statistics of English language knowledge.

Kazdin and Blase [5] noted that smartphones would expand mental health care beyond conventional psychotherapy to previously neglected groups. There is great potential for mobile communication devices to use text messaging and visual images to improve care. The authors noted that current applications are immature. The increasing processing power of smartphones will greatly amplify their potential in the future. Similarly, Rabbitt et al. [6] identified smartphones as a challenging innovation in mental health care delivery. Unfortunately, these researchers are limited by Internet accessibility and spoken language. Even living in a “global village,” the language divide remains an unsolved problem. There are approximately 7.4 billion global citizens [7] who own 6.8 billion cell phones [8]. In addition, two billion [9] of those phones are smart devices (excluding tablets and smart watches). The number of smartphones is predicted to rise to four billion by 2018 [10]. By 2020, almost every registered phone will be a smart device [10]. In comparison, there are only one billion personal computers (PCs) worldwide. Despite the enormous number of people with technological access and skills, many are excluded from communicating globally due to the language divide. According to Anderson et al. [11], over 6,000 languages [12] are used globally, and there is no universal spoken language for communication. The English language is only the third most popular, used by only 5.52% of the global population; Spanish (5.85%) and Mandarin (14.1%) are more common [13]. Moreover, fewer than 40% of citizens of the European Union (not including developing or Eastern countries) know English, which makes communication a problem even within the EU [14] (see Figure 1).

Although technology is progressing exponentially, automatic translation services remain dependent on networks. They require enormous computing power, and their statistical foundation cannot prevent errors. In medical aid, mistakes are not acceptable. Much research is required before voice recognition, machine translation, or text-to-speech (TTS) systems are adequate for medical purposes without the help of a human moderator.

As an alternative, we propose a universal language of pictograms. Obviously, it will not completely replace normal spoken communication. However, we believe that pictograms may be the only method of fast, efficient communication between a patient and a physician in some cases. Furthermore, pictograms have already proven efficient in the communication of people with cognitive disabilities. This study presents a prototype communication aid.

## 2. Review of Current Technology

The need for communication aids by people with auditory disabilities or hearing impairment is well established. An Australian government report [15] identifies three categories of existing technologies that aid such individuals via phones or the Internet: listening devices, text-based technology, and web-based video conferencing technology. Voice-based aids, such as listening devices, are limited to voice amplification and clarification, reducing the number of potential users benefiting from them. Noblin et al. [16] determined that elderly people were reluctant to use telepsychiatry services due to their hearing impairments.

Text-based technologies include TTS, voice recognition, and telephone typewriter (TTY) devices. Special devices are required for older TTY technology. TTS and voice recognition technologies have improved in the past few decades, and some communication aids based on these technologies are currently available. For example, the Virtual Voice app for Android smartphones [17] uses TTS and speech recognition to enable users with hearing impairments to communicate with others by phone without requiring sign language or lip reading. Users with hearing impairments type the text they wish to communicate, and the app uses the smartphone's TTS capability to speak. In the other direction, the app uses the phone's voice recognition capability to translate a caller's voice into text for the user. The app provides large, readable text and supports any language for which a smartphone is configured. In addition, some companies also offer a human voice-to-text translation service. The Hamilton CapTel App for Smartphones (Relay) uses human intermediaries at a call center to translate a voice caller's words into text captions for users with hearing impairments. The captions are displayed on the user's phone. These text-based communication aids depend on a user with adequate cognitive abilities and on a human intermediary.

Web-based video conferencing is sometimes used by people with auditory disabilities, especially by those who use sign language [15]. A video relay service (VRS) enables users with hearing impairment to use American Sign Language (ASL) with a human communications assistant (CA) via video (Commission). The CA then relays information between the user with hearing impairments and a voice telephone user. Auslan Services provides a human VRS over Skype. Purple Communications offers the P3 mobile app and PC software, which supports VRS for iPhones and Android smartphones (Commission). Sorenson Communications offers a similar app and service called ntouch (Communications). There are other such services offered by other vendors. Some governments also offer similar services, such as the National Relay Service (NRS) in Australia (Service). However, the NRS is mostly available via TTY devices.

Considerable research has been conducted on gesture recognition and translation (e.g., ASL gestures). However, gesture recognition requires special equipment (e.g., video cameras or a glove that is worn by the gesturing user), supports only one-way communication, and relies on error-prone statistical models. Foong et al. [17] developed a prototype “Sign to Voice” system that automatically recognizes universal hand gestures using neural networks and translates the signs into a voice output. Vutinuntakasame et al. [18] presented a system that recognizes gestures and translates them into sounds. However, the validated system capabilities were quite limited. More recently, the Computer and Automation Research Institute in Hungary developed the InterpreterGlove [19, 20], which translates sign language gestures into voice. The InterpreterGlove assists sign language users in communicating with others [21]. A student in India developed a digital glove that enables people with vocal impairments to orally communicate with people by translating gestures into voice and text [22]. Bhat et al. [23] developed a technology to translate different gestures, including Indian sign language, to text and voice using flex sensors placed on hand gloves in conjunction with Bluetooth wireless networks and cell phones. This technology is intended to aid patients who have speech or hearing impairments or who are paralyzed.

Several researchers have recognized the potential for using pictograms in smartphone-based communication for medical applications and disadvantaged users. For example, the Talk Up! Application [24] supports the use of pictograms for communication on Android tablets and smartphones. It was designed for children with difficulties in verbal communication (e.g., autistic children). The software comes with a basic set of thematically grouped pictograms to which users can append. The user can assemble a series of pictograms. Sounds associated with the pictograms (e.g., words) can then be played by the software [25].

Proloquo2Go is a commercial mobile app that enables users with vocal impairments to communicate with others using pictograms [26]. It runs on the Apple Watch, iPhone, and iPad. The app is currently only available in English. A user can create a series of pictograms and produce the sounds of words or expressions associated with the pictograms using TTS.

The iPicto app [27], which runs on the iPhone and iPad, enables users with cognitive disabilities to communicate with other people using pictograms. Textual words can be mixed with pictograms and sent via email.

There are some issues with using these approaches in emergency medical communication for people with hearing, speech, or cognitive impairments. Listening devices for speech enhancement rely on some level of hearing ability. Traditional TTY requires the user to have special devices available. Other text-based communication approaches require high cognitive ability. Various web-based video conferencing systems rely on hearing, speech, or knowledge of sign language. VRS and some text-based communication services require a human intermediary. In addition, text-based communication can be slow. These disadvantages make these approaches unsuitable for the medical domain.

Speech recognition, voice synthesis, and gesture recognition depend on error-prone statistical models, unsuitable for emergency medical communication. Gesture recognition is also dependent on gesture quality and, in the case of video-based recognition, image quality. Glove-based gesture recognition requires the use of a special device. Some approaches require the user to know sign language.

In contrast, pictogram-based communication systems have been successfully implemented in other domains and for usage by children. These systems have proven to be more accurate in some cases as cross-lingual and cross-cultural means of communication. Researchers in [28] developed a set of 550 pictograms for foreign Japanese students and conducted successful experiments on cross-lingual communication using their system. The meaning was fully understood by 83% of people chatting only using pictograms. The same research group also conducted research on cross-lingual differences within pictogram-based chat systems [29]. They closely examined pictogram taxonomy and selection. Experiments were carried out nine times in international conferences in America, Vietnam, and Portugal between people who do not share the same spoken language, native language, or culture. The results indicate that the level of understanding was 91.1%. Pictogram communication was enough to make major advances in communication.

The authors of [30] focused on a pictogram-based instant messaging service intended to bridge social and digital gaps among people with cognitive impairments. By using pictograms as the communication language and by tailoring the interface to suit pictogram-based communication requirements, the service allows users to exchange real-time messages across the Internet to communicate with their relatives and acquaintances. Initial research with a group of eleven users with different types and degrees of cognitive impairments showed that a pictogram-based instant messaging service has great potential to improve communication and enable personal and social development. However, their system was limited to cognitive impairments, and the evaluation group was very small.

PictNet is an online pictogram communication system designed for children from different countries to communicate with multilingual support while reflecting each user's cultural background. Online pictogram surveys and experimental pictogram creation activities for children indicate that users can search for pictograms more easily when the pictogram repository has a semantics framework of not only the relationships among concepts in PictNet but also relationships among the visual properties of pictograms and the cultural and emotional context of pictograms [31].

In summary, a few pictogram-based systems have been developed. However, current systems are often complex (by trying to be a universal means of communication), combining textual words and symbols or a complicated set of symbols and thus making perception harder and slower for mentally disabled people or people with eye problems. Other pictogram approaches translate symbols to output text or sounds. This delays communication (clear, intuitive pictures are processed faster by humans) and may require one or both users to have normal hearing ability. Furthermore, we found no pictogram-based communication systems designed specifically for medical applications. Pictogram-based communication appears promising for use by individuals with speech, hearing, or cognitive impairments. Visual symbols can be an intuitive way of communicating in a way that is independent of the user's language, country of origin, and the person with whom they are communicating. Pictograms do not require knowledge of a specialized language (e.g., ASL), specialized equipment (e.g., TTY device or a digital glove), or a human translator (e.g., as in VRS). If the design is simple and only symbols are used, then such pictogram-based communication may provide many benefits. In this research, we propose a pictogram-based communicator designed for but not limited to a smartwatch. Using a smartwatch is faster and more convenient than using a tablet or phone. In addition, the device is fastened to person's hand and allows medical personnel to quickly collect information in an emergency situation even if the patient is unconscious. Our prototype is limited to the first aid domain and makes a small number of pictograms faster to process by humans, especially disabled people. The prototype also makes human-device interaction as intuitive as possible.

## 3. Patient-Physician Communicator Prototype

To explore pictogram-based communication between patients and physicians, we developed a conceptual prototype and conducted usability and accessibility tests to refine its design [32]. Our prototype is a smart watch application (also usable with smartphones and tablets) that aids nonnative language speakers or people with speech, hearing, or cognitive impairments to communicate relevant medical information to physicians. It provides simple, clear communication by using intuitive icons and interactive symbols. They are easy to find on the smart watch, which is easy to access and difficult to misplace.

Such an application would have clear benefits for many users. For example, a person with cognitive disabilities or impairments could very quickly and easily use simple icons to convey basic information about injuries, emotions, pain level, or basic needs to medical personnel. Similarly, medical personnel could use this application to assist someone who is injured or paralyzed. Quick, simple, easy, and intuitive communication is needed, especially in emergency medical situations.

**Figure 2:**
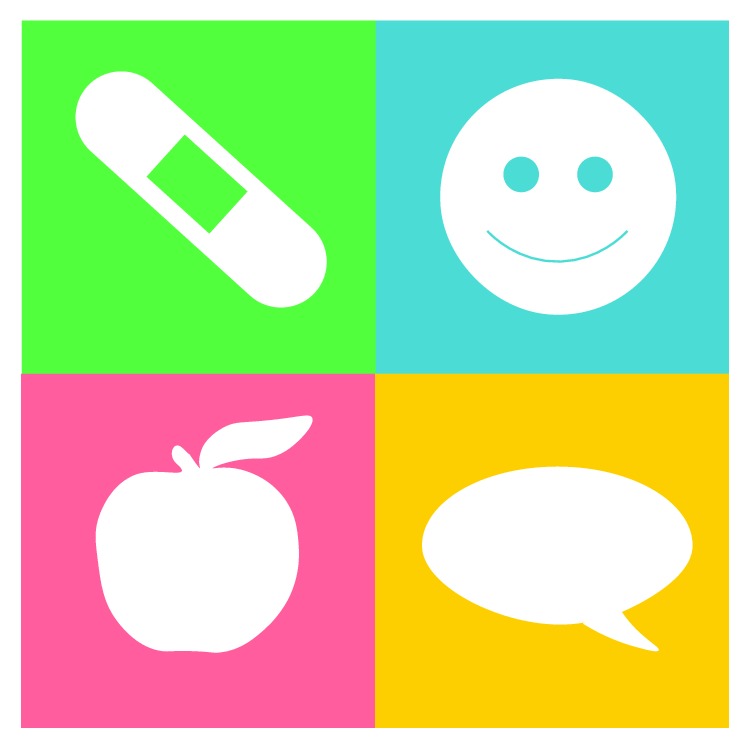
Basic smart watch application parts.

An iterative paper prototype was developed and evaluated in accordance with the process described by Albert and Tullis [33] to design the application. This approach creates rough screen mock-ups or hand sketches that are evaluated by users. Such early usability and accessibility tests helped develop a design for our patient-physician communicator prototype [34]. The application has four basic parts, shown in Figure 2, to communicate a need for medical aid (band-aid), moods or emotions (smiley face), diet and allergy-related issues (apple), and a chat function (call-out balloon). These pictograms provide a very simple, easy-to-use interface. Each application feature has a pictorial presentation that uses images serving as intuitive symbols for various functions. The application also uses a very simple, attractive layout with a cool color scheme (avoiding eyestrain) and is highly accessible for people with visual problems.

**Figure 3:**
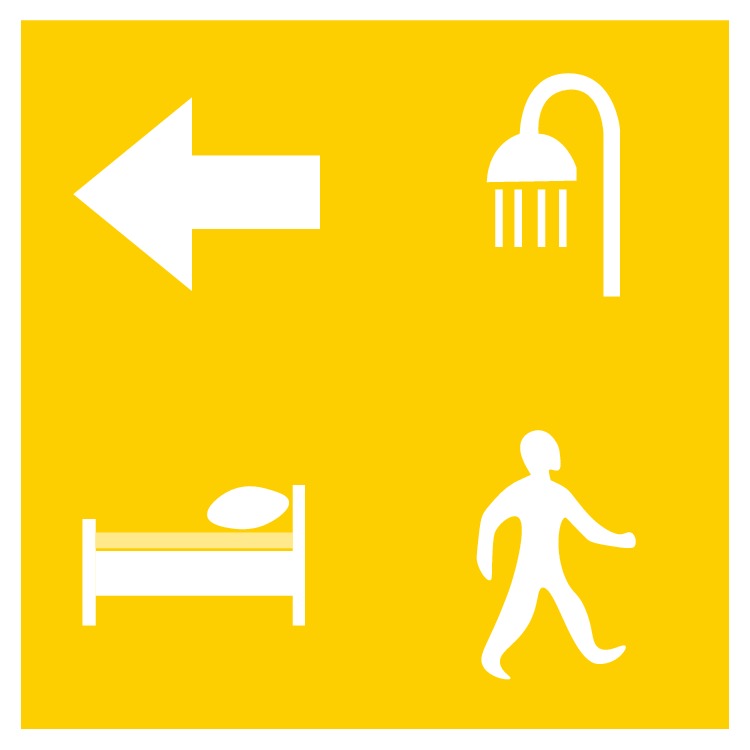
Navigation and additional functions.

Simple application navigation symbols, such as a back arrow, enable users to move easily through the application to find their target content. Navigation and additional functions are shown in Figure 3. The other symbols in this figure indicate that the user needs a bath (shower), rest (bed), or exercise (person walking).

**Figure 4:**
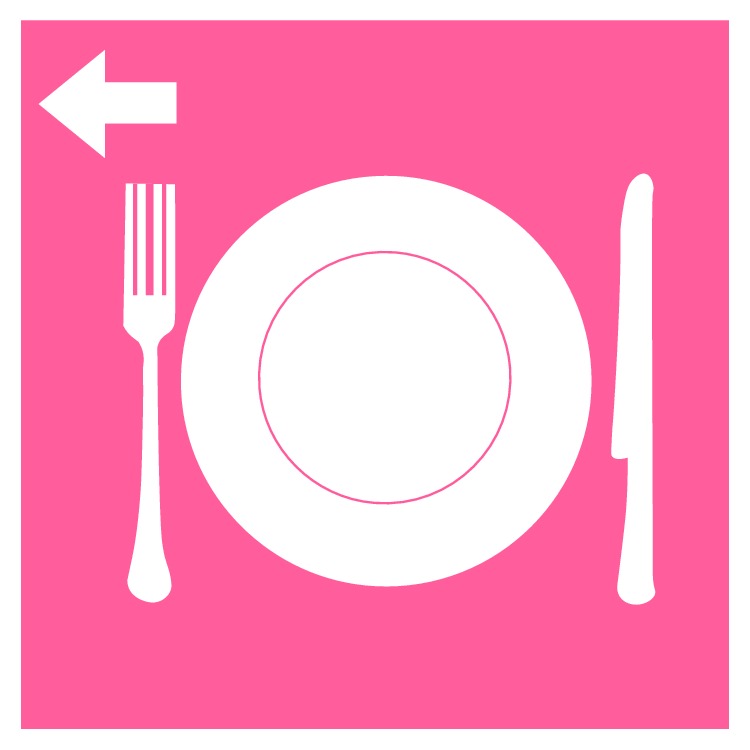
Diet symbol for hunger.

Figure 4 shows an example screen when the user chooses the diet function. This symbol indicates that the user is hungry and needs to eat. Other symbols might be used to indicate allergies or other diet-related issues.

**Figure 5:**
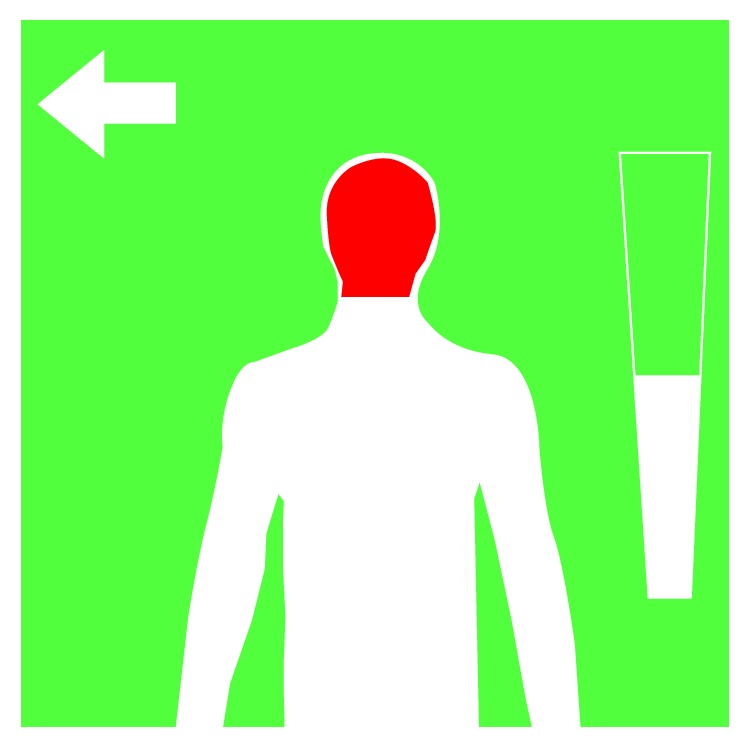
Aid to communicate pain.

Figure 5 shows a screen that enables a person to indicate specifics about their pain experience. The user can select the part of their body experiencing pain. They can also select pain level. These are common methods medical personnel currently use to elicit pain information from patients.

**Figure 6:**
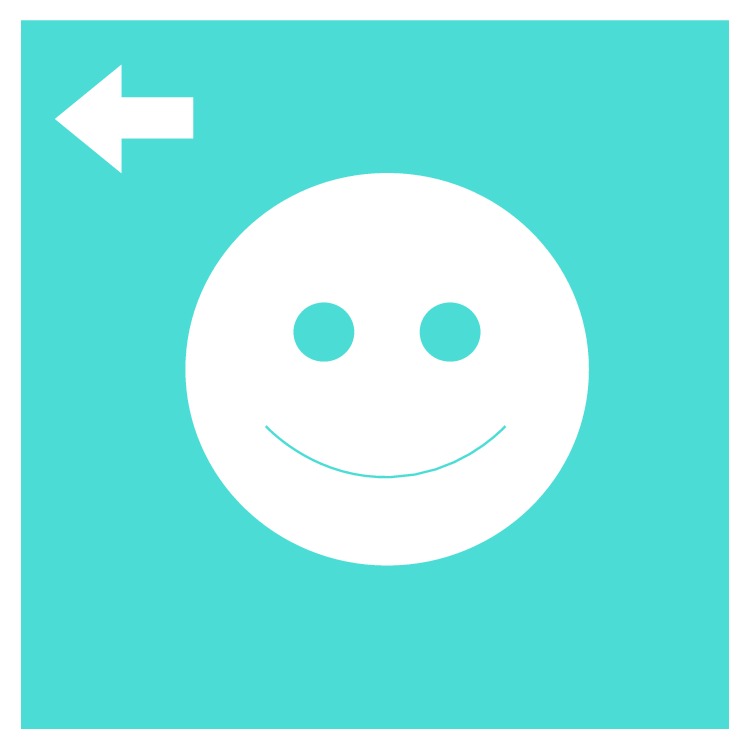
Aid to communicate mood or emotion.

A screen to indicate mood or emotion is shown in Figure 6. Users can select an emoticon (pervasive in the Internet-centric world today) to communicate this information to medical personnel.

## 4. Results

A preliminary usability review of the patient-physician communicator prototype was conducted online with 50 random people of different ages and genders. To find participants, we used services of the Ankieka.pl portal. We did not use acceptance or exclusion criteria, so every person or their caregiver could take part in the experiment. However, it was necessary to provide general information about disability. The Visual Simulations [35] and Fujitsu Color Selector [36] tools ensured that the application design would be accessible to almost everyone. In a participant questionnaire, we asked for sex, nationality, age, and disabilities. We asked participants to complete three tasks. In task 1, participants had to use our prototype to indicate that they require rest. In task 2, they had to indicate medium pain in their left leg. Lastly, in task 3, they had to indicate that they were hungry. After each task, the prototype determined whether or not the task was successfully completed, measured the time required for each task, and asked participants for their subjective feelings about the difficulty and intuitiveness of the task. The application worked well. We summarize the feedback below.

**Figure 7:**
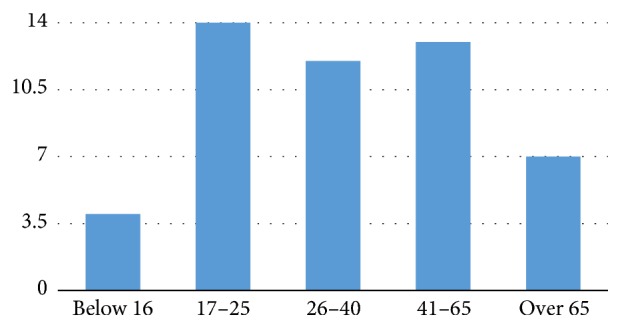
Age groups statistics.

Of the 50 participants, 56% were male. The lowest percentage of all age groups was the <16 years old category. The most prevalent age category was the 17–25 years old group, as shown in Figure 7.

**Figure 8:**
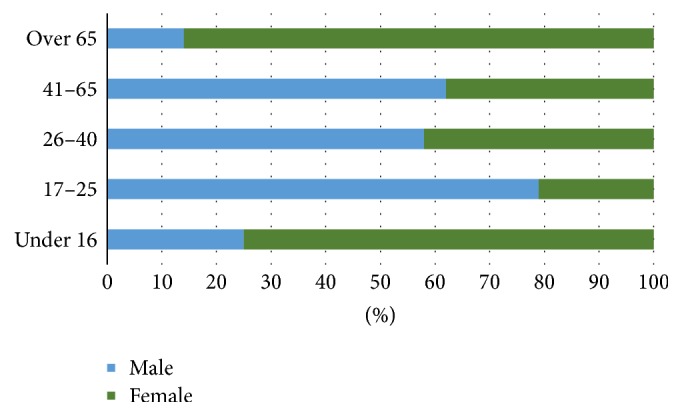
Gender by age.

In the most prevalent age group, 79% were male. The highest female representation was within the >65 age category, in which 86% were female (Figure 8).

**Figure 9:**
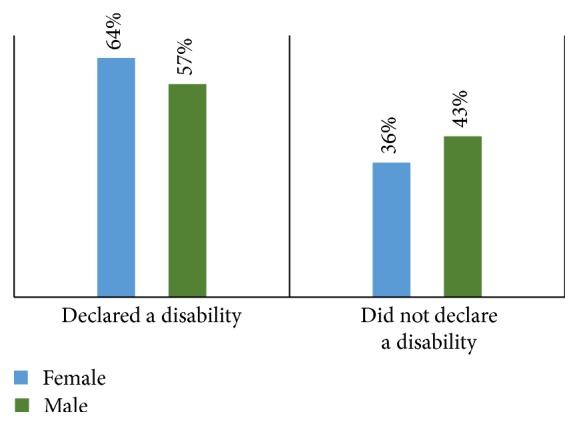
Disability by gender.

The largest nationality represented was Polish, 40% of all users. Consistent with this, Poland led the majority percentage within each age category. Sixty percent of all users declared a disability; 57% of all males and 64% of all females stated that they had a disability (Figure 9).

**Figure 10:**
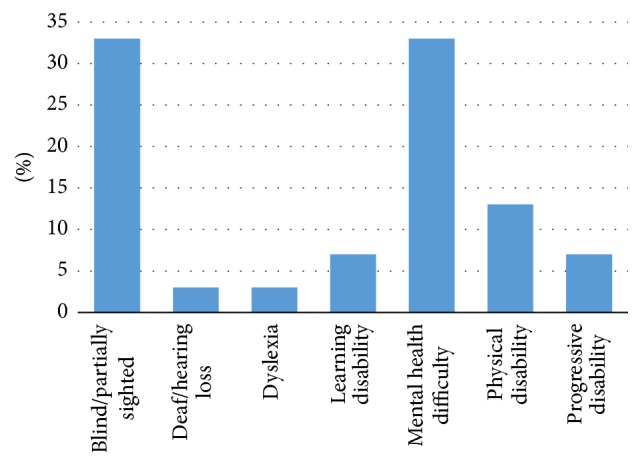
Disability breakdown.

**Figure 11:**
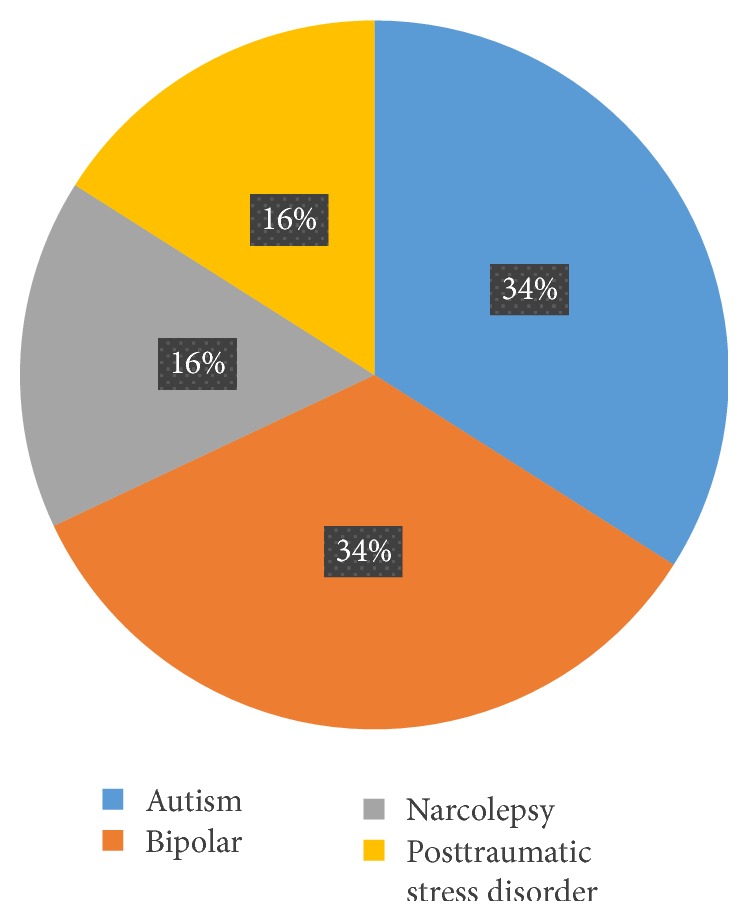
Mental health.

**Figure 12:**
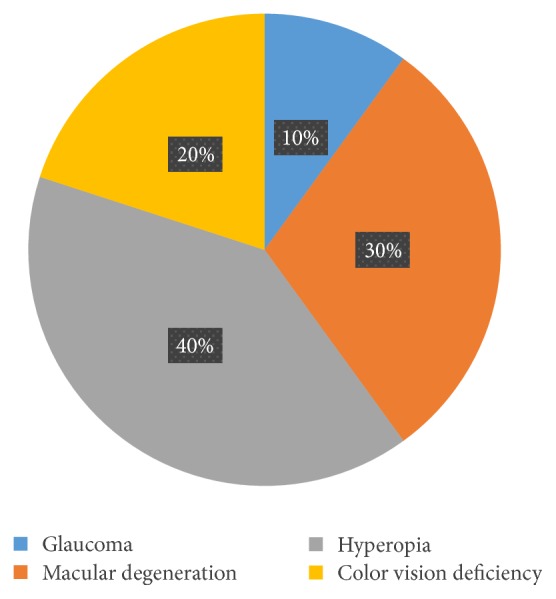
Visual impairment.

The highest percentage of disability declaration was the <16 age category, 75%; this was followed by the >65 categories with 71%. The healthiest age group was 26–40 years, in which 58% noted no disability. Among the 40% Polish users, 50% stated they did not have a disability. Of non-Polish users, 20% declared they did not have a disability. The highest percentages of disabilities were the “mental health difficulty” and “blind/partially sighted” categories, each with 33%. Participants with mental health disabilities, autism, and bipolar disorder were the most prevalent, with 34% each. Among people with visual impairments, 40% had hyperopia and 30% had macular degeneration and 20% had color vision deficiencies, of which protanopia and deuteranopia each were 50% (Figures 10, 11, and 12).

Of all users, 96% were able to successfully complete the three assigned tasks; 92% completed the tasks without difficulty. All (100%) failed tasks were performed by users with learning disabilities. With all three tasks, the highest percentage of time to completion was 31–60 seconds (Tables 1, 2, and 3).

On a scale of 1–5 where five represents very easy, the highest percentages of each task were rated either 4 or 5 by significant margins. Four additional statements were provided to the user on similar scales on which one represented strongly disagree and five represented strongly agree. When asked if the user immediately understood the function of each button, 50% rated this aspect a 4, and 42% rated it a 5. The second statement was “the buttons were well organized and easy to find.” Ratings 4 and 5 each obtained 48%. The third statement was “the application was easy to navigate.” A rating of 5 was the most prevalent (66%), while 28% of participants selected a rating of 4. The final statement was “my overall impression of the application is positive.” Once again, 66% of users strongly agreed with this; 32% rated it at 4.

Four additional questions were asked to users. These were on a scale of 1–5, where 1 represents “strongly disagree,” and five represents “strongly agree” (Tables 4 and 5).

- Statement 1—I immediately understood the function of each button.
- Statement 2—The buttons were well organized and easy to find.
- Statement 3—The application was easy to navigate.
- Statement 4—My overall impression of the application is positive.

## 5. Discussion and Conclusions

Enabling people with language difficulties or disabilities to communicate medical information to physicians and other medical personnel is a considerable challenge. The communication must be quick, accurate, simple, and intuitive. Aids for such medical communication are lacking. Existing solutions for general use have some disadvantages. They often require some hearing or high cognitive ability. Some require special devices or human intermediaries. Many are slow means of communication. Some approaches depend on imperfect error-prone statistical models. Current pictogram-based approaches are complex and have many of the same drawbacks; most importantly, they are not adapted for medical purposes.

Our proposed approach leverages the power of visual communication. It is fast, simple, intuitive, and designed specifically for emergency medical applications. No knowledge of or skill in sign language or gesturing is needed, nor are speech, hearing, or high cognitive abilities required. A preliminary usability review of our prototype has demonstrated the potential of this approach.

In conclusion, the application worked well regardless of age, gender, nationality, or disability. No correlation of concern or correction was present. The overall feedback from all users was very positive. Application availability on smart watches increased its accessibility. Users had easy access to it at any moment; because a smart watch is attached to the person's wrist, it is more difficult to lose and less cumbersome to access than a smartphone. Such applications can also contain medically relevant information in readable text for a physician. If a smart watch has appropriate sensors, automatic communication would be possible if the patient has collapsed. The visual communication technique of using pictograms appears to be a good one, as it is simple, direct, and intuitive for users with speech, hearing, or cognitive impairments. People can very quickly interpret good visual communication. “A picture is worth a thousand words” is an established maxim in human experience that has been validated by research. Visual images are processed 60,000 times faster than text [37]. Graphics also quickly affect our emotions, and emotions greatly affect our decision-making [38].

A future direction for our research is an extension of the application's capabilities in collaboration with medical personnel. In addition, we envision conducting usability testing with medical personnel and people with speech, hearing, and cognitive impairments to assess and improve the design. Lastly, pictogram analysis is necessary to ensure that they are easily understood and similarly interpreted across different cultures.

## Figures and Tables

**Table 1:** Task 1 rating versus disability.

	1	2	3	4	5
Blind/partially sighted	0%	0%	0%	60%	40%
Deaf/hearing loss	0%	0%	0%	100%	0%
Dyslexia	0%	0%	0%	100%	0%
Learning disability	50%	50%	0%	0%	0%
Mental health difficulty	0%	0%	0%	90%	10%
Physical disability	0%	0%	0%	25%	75%
Progressive disability	0%	0%	0%	50%	50%

**Table 2:** Task 2 rating versus disability.

	1	2	3	4	5
Blind/partially sighted	0%	10%	0%	70%	20%
Deaf/hearing loss	0%	0%	0%	0%	100%
Dyslexia	0%	0%	0%	100%	0%
Learning disability	0%	50%	50%	0%	0%
Mental health difficulty	0%	0%	10%	40%	50%
Physical disability	0%	0%	0%	0%	100%
Progressive disability	0%	7%	7%	43%	43%

**Table 3:** Task 3 rating versus disability.

	1	2	3	4	5
Blind/partially sighted	10%	0%	0%	50%	40%
Deaf/hearing loss	0%	0%	0%	0%	100%
Dyslexia	0%	0%	0%	100%	0%
Learning disability	50%	0%	0%	0%	50%
Mental health difficulty	0%	0%	10%	60%	30%
Physical disability	0%	0%	0%	50%	50%
Progressive disability	0%	0%	0%	100%	0%

**Table 4:** Statement 1 and statement 2 versus disability.

Statement 1						Statement 2					
	1	2	3	4	5		1	2	3	4	5
Blind/partially sighted	0%	0%	0%	30%	70%	Blind/partially sighted	0%	0%	0%	60%	40%
Deaf/hearing loss	0%	0%	0%	0%	100%	Deaf/hearing loss	0%	0%	0%	0%	100%
Dyslexia	0%	0%	0%	100%	0%	Dyslexia	0%	0%	0%	100%	0%
Learning disability	0%	0%	50%	50%	0%	Learning disability	0%	50%	50%	0%	0%
Mental health difficulty	0%	0%	0%	60%	40%	Mental health difficulty	0%	0%	10%	60%	30%
Physical disability	0%	0%	0%	25%	75%	Physical disability	0%	0%	0%	25%	75%
Progressive disability	0%	0%	0%	0%	100%	Progressive disability	0%	0%	50%	0%	50%

**Table 5:** Statement 3 and statement 4 versus disability.

Statement 3						Statement 4					
	1	2	3	4	5		1	2	3	4	5
Blind/partially sighted	0%	0%	0%	50%	50%	Blind/partially sighted	0%	0%	10%	40%	50%
Deaf/hearing loss	0%	0%	0%	0%	100%	Deaf/hearing loss	0%	0%	0%	0%	100%
Dyslexia	0%	0%	0%	100%	0%	Dyslexia	0%	0%	0%	0%	100%
Learning disability	0%	0%	100%	0%	0%	Learning disability	0%	0%	100%	0%	0%
Mental health difficulty	0%	0%	0%	70%	30%	Mental health difficulty	0%	0%	0%	30%	70%
Physical disability	0%	0%	0%	25%	75%	Physical disability	0%	0%	0%	0%	100%
Progressive disability	0%	0%	0%	100%	0%	Progressive disability	0%	0%	0%	100%	0%
